# The mitochondrial genome of *Gymnopternus bomiensis* (Diptera: Dolichopodidae)

**DOI:** 10.1080/23802359.2022.2090294

**Published:** 2022-07-04

**Authors:** Qilemoge, Ding Yang

**Affiliations:** a School of Biological Science and Technology, Baotou Teachers’ College, Baotou, China; b College of Plant Protection, China Agricultural University, Beijing, China

**Keywords:** Mitochondrial genome, *Gymnopternus*, phylogenetics

## Abstract

The mitochondrial genome of *Gymnopternus bomiensis* (Yang, 1996) (Diptera: Dolichopodidae) has been reported in this study. This is the first mitogenome representative of *Gymnopternus*. The sequenced region is determined to be 15,212 bp, including 13 protein-coding genes, 2 ribosomal RNAs, 22 transfer RNAs, and a partial A-T rich region. The nucleotide composition biased toward A and T, and the overall A + T% was up to 74.0％. Additionally, we reconstructed the phylogeny of relative species using 13 PCRs and two rRNAs. Bayesian inference analysis strongly supported the monophyly of Dolichopodinae. It also suggested that *Gymnopternus* is the sister group of *Dolichopus*.

The *Gymnopternus* Loew, 1857 is a genus of Dolichopodinae with 133 species in the world (Yang et al. 2006; Yang et al. 2011; Grichanov 2017).

The adult specimens of *Gymnopternus bomiensis* (Genbank accession number: OK298480) were collected from Zhouzhi (37°16′93″ N, 108°22′87″ E, 1740 m) of Shaanxi Province in China by Xuankun Li on 1 August 2015. The specimens were deposited in the Entomological Museum of China Agricultural University (Voucher Number: D-GYM-1; Liang Wang, 1352659341@qq.com). The total genomic DNA was extracted from the whole body of a male specimen using the QIAamp DNA Blood Mini Kit (Qiagen, Germany) and stored at −20 °C. The sequencing was followed the procedures of Gillett et al. (2014), the pooled dsDNA sample was sent to BIONONA CO., LTD (San Diego, CA) for library construction and sequenced by the Illumina HiSeq 2500 platform (Illumina, San Diego, CA). The final filtered reads were assembled with Meta-IDBA (Peng et al. 2012). The circularization was checked using circle_check. py in MitoZ software (Meng et al. 2019). The nearly complete mitogenome of *G. bomiensis* is 15,212 bp. The A-T rich region could not be sequenced entirely, the length of fragment is 556 bp, but the neighboring gene sequences were completed. It included all coding regions, 13 protein-coding genes, 22 transfer RNAs, and 2 ribosomal RNAs, which was qualified for most of analysis related to mitochondrial genomes as reported before (Wang et al. 2016; Qilemoge, Gao, et al. 2018; Qilemoge, Zhang, et al. 2018; Hou et al. 2019; Qilemoge, Zhang, et al. 2019; Qilemoge, Lin, et al. 2019; Lin and Yang 2021; Wang et al. 2021). The nucleotide composition of *G. bomiensis* mitochondrial genome was biased toward A and T (A = 39.0%, T = 35.0%, C = 15.5%, G = 10.5%). The A + T content of protein-coding genes, transfer RNAs, ribosomal RNAs was 72.2%, 75.9%, and 77.9%, respectively. The total length of all 13 PCGs was 11,201 bp. All PCGs of *G. bomiensis* utilized the conventional start codons for invertebrate mtDNA. *COII*, *COIII*, *ATP6*, *ND4, ND4L*, and *CYTB* initiated with ATG codon; *ND2*, *ND3*, *ND5*, *ND6*, and *ND1* initiated with ATT codon; *ATP8* initiated with ATA codon; while *COI* initiated with TCG codon, which is a common start codon for insect *COI* gene. Ten PCGs used the typical termination codons TAA, and the remaining three, *CytB*, *ND1*, and *ND3*, used TAG codon.

Phylogenetic analysis was performed based on 13 PCGs and 2 rRNAs of 12 related species. One Asilidae species (*Leptogaster longicauda*) was chosen as the outgroup, and two Empididae species was supplemented considering the close relationship between Empididea and Dolichopodidae. All available Dolichopodidae data were added. Bayesian (BI) analysis (Figure 1) showed that monophyletic Empididae was sister to monophyletic Dolichopodidae. The phylogenetic relationship of Dolichopodidae was (Hydrophorinae + Sympycninae) + (Dolichopodinae + (Sciapodinae + (Diaphorinae + (Medeterinae + Rhaphiinae)))). The *Gymnopternus* was assigned to the sister of *Dolichopus*. The mitogenome of *G. bomiensis* could provide the important information for the further studies of Dolichopodidae or Diptera phylogeny.

**Figure 1.**
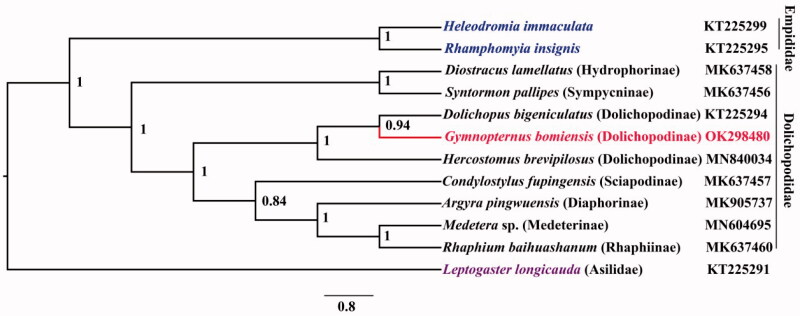
The phylogenetic tree of Bayesian interface analysis based on 13 PCGs and 2 rRNAs.

## Ethical approval

The study protocol was approved by Ethics Committee of Baotou Teachers’ College. This study obtained the field collection permit from Nature Reserve Management Committee of Zhouzhi. The collected insect samples are not protected endangered species.

## Data Availability

The genome sequence data that support the findings of this study are openly available in GenBank of NCBI at https://www.ncbi.nlm.nih.gov under the Accession no. OK298480. The associated BioProject, SRA, and Bio-Sample numbers are PRJNA777443, SRR16889717, and SAMN22860967, respectively.
